# Longer looking to agent with false belief at 7 but not 6 months of age

**DOI:** 10.1002/icd.2263

**Published:** 2021-08-01

**Authors:** Amy Hirshkowitz, M.D. Rutherford

**Affiliations:** ^1^ Psychology, Neuroscience & Behaviour McMaster University Hamilton Canada

**Keywords:** beliefs, eye tracking, infancy, social cognition, theory of mind

## Abstract

Theory of mind refers to the ability to reason about others' beliefs and to understand others' behaviour in terms of those beliefs. A large body of previous research has examined theory of mind reasoning in young children using false belief tasks, but tasks to examine this capacity during infancy have only been developed more recently. This research used stimuli developed by Kovács, et al., *Science*, 2010, 330, 1830–1834, to measure looking time to an agent with a false belief in infants aged 6 and 7 months. Using an eye‐tracking procedure, we found looking behaviour consistent with 7‐month‐olds distinguishing an agent who has a false belief from one who has a true belief, consistent with the results reported by Kovács et al. We did not find evidence of this looking preference among 6‐month‐olds.

**Highlights**

- Eyetracking was used in an implicit false belief task with 6 and 7 month‐old infants.
- Infants saw a false‐belief display, in which the location of a ball is known or unknown to a character.
- Evidence of theory mind found in 7 month‐olds but not 6 month‐olds.

## 1 INTRODUCTION

The ability to reason about others' beliefs and mental states is termed theory of mind (Premack & Woodruff, 1978). An understanding of others' mental states, including beliefs, desires, and goals, is believed to allow one to understand and to predict others' behaviour (Baron‐Cohen, Leslie, & Frith, 1985).

In the last 35 years, the development of theory of mind was measured via explicit responses to false belief (FB) tasks (Baron‐Cohen et al., 1985; Premack & Woodruff, 1978; Wimmer & Perner, 1983). FB tasks typically require children to predict the behaviour of a character who has a FB (Baron‐Cohen et al., 1985; Wimmer & Perner, 1983) or to predict the belief of a character who has only partial knowledge of a situation (Perner, Frith, Leslie, & Leekam, 1989). Given such tasks that require an explicit response to a verbal query, children typically show evidence of theory of mind development by 4 years of age (see Wellman, Cross, & Watson, 2001 for a meta‐analysis).

More recently, implicit FB tasks that rely on looking time as a dependent variable have shown evidence of theory of mind reasoning in younger children and infants. Onishi and Baillargeon (2005) measured 15‐month‐old infants' looking time using a violation‐of‐expectation (VOE) task. In VOE tasks, there are typically two phases: first, the infant is familiarized with the scene, and second, the infant sees one of two outcomes, either surprising or unsurprising given the infants' expectations. Infants' looking behaviour is used as the outcome measure in these tasks. In Onishi & Baillargeon's, 2005 study, using three familiarization trials, an experimenter familiarized an infant with the scenario by reaching for a toy in a number of locations. Then, during the test trial, the infant saw either a true belief (TB) scenario or a FB scenario: in TB test trials, the experimenter knew the location of the toy; in FB test trials, the toy had been moved while the experimenter was absent. The experimenter reached either into the box containing the toy or into the empty box. Looking time data differed significantly between TB and FB test trials: infants looked longer when (a) the experimenter reached to the incorrect location (the empty box) in the TB trials and (b) when the experimenter reached to the correct location (the box containing the toy) in the FB trials. These results suggested that infants tracked the experimenter's belief and expected her to act accordingly (Onishi and Baillargeon (2005).

Over the next decade, tests measuring looking direction or other nonverbal responses continued to produce the evidence of early theory of mind development. One study revealed that 25‐month‐olds show anticipatory looking to locations in which another person falsely believes that there are toys (Southgate, Senju, & Csibra, 2007). Using a violation‐of‐expectation paradigm, Surian, Caldi, and Sperber (2007) found evidence that 13‐month‐olds could use the height of an occluder to determine whether an agent had the visual access necessary to see a preferred food item, and thus form an expectation about which location the agent would approach. Seventeen‐month‐olds were found to use an experimenter's (true or false) belief to track which of two hidden objects an experimenter asked for (Southgate, Chevallier, & Csibra, 2010). Finally, eighteen‐month‐olds were shown to help an adult experimenter achieve her preferred goal when the infant understood that the experimenter had a FB about an object's identity (Buttelmann, Suhrke, & Buttelmann, 2015).

To our knowledge, only one behavioural study has found evidence of FB reasoning with infants as young as 7 months of age (but see Hyde, Simon, Ting, & Nikolaeva, 2018 and Southgate & Vernetti, 2014 for relevant neuroimaging studies). Kovács, Téglás, and Endress (2010) showed 7‐month‐old infants 18.4‐second animated displays in which a blue Smurf‐like character watched a ball roll across a table and behind an occluder. In two familiarization trials, infants viewed the character watching the ball roll behind the occluder and then saw the occluder drop to reveal the ball behind it. Infants then viewed both a TB and FB test trial. In each test trial, the agent walked off‐screen and returned. In TB test trials, the agent watched the ball roll outside the scene to an unseen location. In FB test trials, the ball rolled behind the occluder in the agent's presence and then to an unseen location during the agent's absence. In all test trials, when the occluder was lowered, infants saw the final outcome of no ball being visible. Infants looked significantly longer to FB trials than to TB trials, suggesting that they tracked the agent's belief regarding the presence or absence of the ball behind the occluder.

One limitation of Kovács et al.'s study is the possibility of retroactive interference. Heyes (2014) argues that infants may have looked longer to the FB trials than to the TB trials not because of belief tracking, but because the return of the agent in FB trials after the ball moved off‐screen interfered with infants' formation of a memory of the ball moving off‐screen. Because this memory of the ball's movement was impaired, FB trials were more similar than TB trials to the familiarization trials, since during familiarization trials, the final outcome revealed the ball when the occluder dropped. From this perspective, the ultimate absence of the ball was surprising, but only in FB trials.

One approach to addressing Heyes's concern is to use eye tracking to measure whether the infant's gaze is directed at the agent or at the area that is revealed once the occluder has dropped at the end of a FB trial. If attention is drawn to the ball (or its absence), then the infant would look longer at the expected location of the ball, the area that had been occluded. Similarly, if the infant is surprised on behalf of the agent, gaze would be not to the agent but to the newly revealed evidence: the ball or its absence. In contrast, only if the surprise that results from the FB of the agent is of interest, would the infant's gaze be directed to the agent. Evidence from adult participants suggests that a model's gaze captures attention more if that model has a surprised facial expression (Bayless, Glover, Taylor, & Itier, 2011; Neath, Nilsen, Gittsovich, & Itier, 2013). We reasoned that if an infant watches as an agent is confronted with evidence challenging a FB, the agent's resulting surprise could attract the attention of the infant.

Kovács and colleagues reported that in studies with 7‐month‐olds, the presence of a character with an apparent FB can impact infants' looking behaviour. Still, it is an open question whether there is any FB reasoning at 6 months of age. There is evidence that 6‐month‐olds view reaching and grasping as goal‐directed (Woodward, 1998). There is even some evidence of social referencing as early as 6 months of age (Mireault et al., 2014). Neuroimaging studies have reported neural responses consistent with an appreciation for FB at 6 months (Hyde et al., 2018; Southgate & Vernetti, 2014). Thus, some components of theory of mind are already developing at this age. However, we know of no behavioural evidence that 6‐month‐olds understand FB displays. This study measures 6‐and 7‐month‐old infants' looking behaviour when a character has a FB versus a TB.

### 1.1 The current study

Kovács et al. (2010) reported greater looking time to displays in which an agent had a FB compared with trials in which an agent had a TB. The purpose of this study was to build on previous findings regarding FB reasoning in 7‐month‐olds by testing whether the infants' looking behaviour was directed toward the agent or to expected location of the ball. In other words, do they attend to different aspects of the scene? We also extended previous findings by testing whether 6‐month‐olds also respond differently to FB and TB trials.

To this end, we used the stimuli provided by Kovács et al. (2010) and made the following changes to their design: (a) in answer to criticism by Heyes (2014) that FB trials were more similar to the familiarization trials than TB were, we did not present infants with familiarization trials. Rather, all infants viewed only eight test trials (4 TB, 4 FB), (b); each infant viewed all TB and FB trials. The final outcomes (ball or no ball) were always congruent with infants' beliefs, but in FB trials, outcomes were incongruent with the agent's beliefs, and (c) trial length was kept constant (rather than infant‐controlled) so that all infants viewed the final outcome for 5 s after the occluder dropped. The use of eye‐tracking technology allowed us to track gaze direction during the final 5 s of trials, and to measure whether infants spend more time looking at the agent who might be surprised or at the surprising evidence: the expected location of the ball. We reasoned that if infants were tracking the character's beliefs, they would look more to the character (expecting the character's surprise) in FB trials than in TB trials. We tested both 6‐ and 7‐month‐old infants to see whether these two age groups would visually explore these scenarios in a similar way.

## 2 METHOD

### 2.1 Participants

Thirty‐four infants were included in analyses (Caucasian = 30, Other/mixed race = 4). This included two age groups with seventeen 6‐month‐old infants (mean age = 6 months, 8 days; age range = 179–195 days; 8 males and 9 females) and seventeen 7‐month‐old infants (mean age = 7 months, 6 days; age range = 208 days‐230 days; 8 males and 9 females). We planned to recruit approximately 20 participants per age group, because we wanted to have a similar sample size to the original study (Kovács et al., 2010; *n* = 14 per age group), while allowing for some loss for fussiness or equipment failure. In order to find an effect of the size that Kovács et al. reported (*d'* = 1.44), we would need to compare two groups with a minimum sample size of 7 per age group (or 14 total). In fact, we were successful in recruiting 43 participants to the laboratory. Nine infants were tested and excluded for fussiness (*n* = 5), technological malfunction (*n* = 2), or autism spectrum disorder (ASD) diagnosis of a close relative (*n* = 2). The latter were excluded since theory of mind development is known to be atypical in children with ASD (Baron‐Cohen et al., 1985). No analyses were started before data collection was complete.

Infants and parents were recruited through hospital visits to local maternity wards, where providing contact information for the purpose of research participation is completely voluntary. Participants were paid $15 for their participation. Informed consent was obtained, and an explanation of experimental procedure was provided prior to testing.

### 2.2 Apparatus and data recording

A remote eye tracker (Tobii T60XL) with infrared corneal reflection technology embedded into the monitor was used to measure eye movements during stimuli presentation. The display monitor was a 24‐in. flat screen set to the resolution of 1,024 × 768 pixels. The Tobii T60XL recorded data at 60 Hz and 0.5° visual angle accuracy. A web camera was placed atop the monitor to record infants' faces in a full‐frontal view. Stimuli were presented with Tobii Studio software using a Dell desktop computer with a Windows 7 operating system.

### 2.3 Stimuli

Four trial types were used. All trials were 24 s. All trials depicted an animated blue character (agent) watching a ball rolling across a table and behind an occluder, and included the agent leaving and returning to the scene. In FB trials, when the agent walked off‐screen, the ball either rolled to a different unseen location after the agent had viewed the ball rolling behind an occluder (no ball trials), or the ball rolled back behind the occluder after the agent had viewed the ball rolling off‐screen (ball trials) in the agent's absence. In TB trials, when the agent walked off‐screen, the ball remained in the same location during the agent's absence. Either the agent viewed the ball rolling to a different unseen location, and the ball remained off‐screen in the agent's absence (no ball trials), or the agent viewed the ball rolling behind the occluder and the ball remained there in the agent's absence (ball trials). In all trials, the agent returned to the scene and the occluder dropped down to reveal the presence or absence of the ball. The trial ended 5 s later. Note that in the original experiment (Kovács et al., 2010), each trial ended with no visible ball, but in this study, half of the trial end with a visible ball, and half the trials end with no visible ball, so that we can test whether looking behaviour is explained by the presence of a ball.

### 2.4 Design

In the current design, age was a between‐subject factor, with 7‐month‐old and 6‐month‐old groups. The Smurf‐like agent's true versus FB was a within‐subject factor since every infant saw four trials in which the agent was present when the ball rolled out of the scene and four trials in which the agent was absent when the ball rolled out of the scene. The infant's TB was constant across these eight trails, as the presence or absence of the ball behind the occluder could always be anticipated by the infant. The final outcome, whether the ball was behind the occluder or not, was also a within‐subject factor. All infants viewed a total of eight 24‐second stimuli trials with agent's belief (4 FB, 4 TB) crossed with the final outcome (4 Ball, 4 No Ball). Trial order was randomized for each participant during the session: at each trail, a trial type was selected randomly without replacement by the eye tracker and displayed as the next trial. Table 1 illustrates this design.

**TABLE 1:** Current study design

	False belief	True belief
Ball present	Infant expected ball Agent surprised by ball	Infant expected ball Agent expected ball
Ball absent	Infant expected absence Agent surprised by absence	Infant expected absence Agent expected absence

### 2.5 Procedure

Infants were seated in a car seat (*n* = 28) or parent's lap (*n* = 6) with eyes 65 cm away from the eye‐tracking monitor. The testing room was dimly lit to reduce distractions. Parents sitting with their infants were asked to close their eyes to avoid looking at the monitor. The Tobii Studio 5‐point infant calibration with animated stimuli was used to obtain reliable eye movement data.

### 2.6 Data coding

**FIGURE 1:**
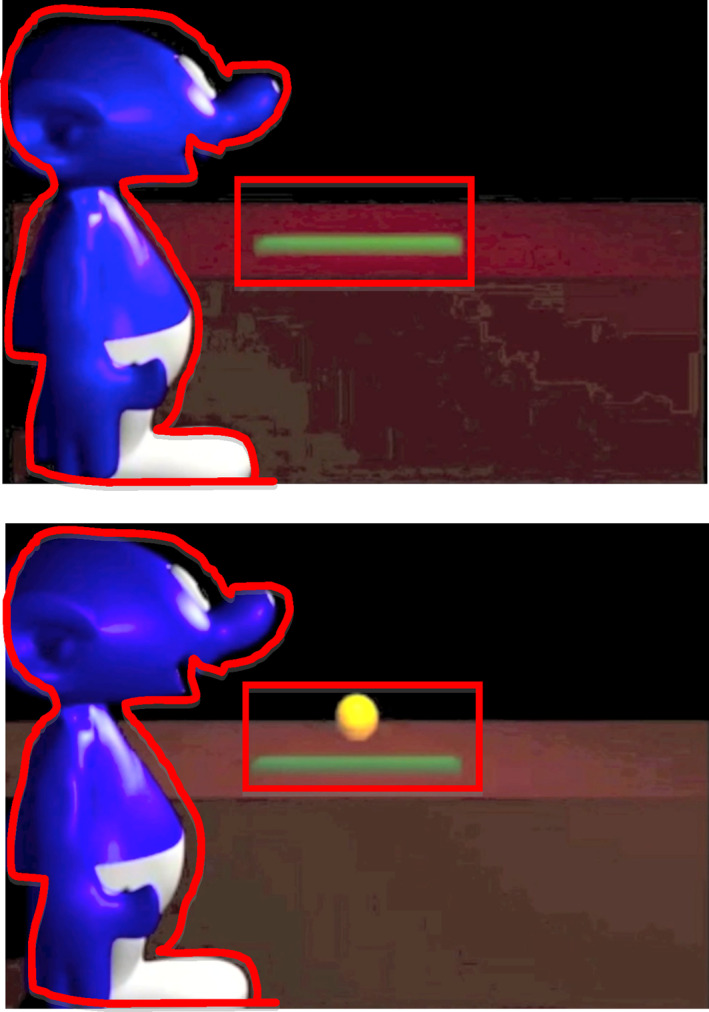
Stimuli used in this study. Final frame of TB and FB trials (5 s each) shown with red areas of interest (AOIs) depicted. Infants viewed four trials total: each trial type (TB, FB) had one trial of each outcome (ball present, ball absent)

Total looking time and total number of fixations to two areas of interest (AOIs) in the final 5 s (occluder down) of each trial were coded. These AOIs isolate the agent or the area that is revealed when the occluder turns down. In order to avoid false positives, the Agent AOI was drawn around the outline of the agent, rather than by creating an oval‐shaped AOI (see Figure 1 and Appendix A). Fixation data were defined using the Tobii fixation filter with a velocity threshold of 35 pixels and a distance threshold of 35 pixels.

## 3 RESULTS

### 3.1 Preliminary analyses

A multivariate 2x2 ANOVA with the between‐subjects factor of *Stimulus Order Viewed* (FB First, TB First) was computed for both the mean total looking time and mean total number of fixations to the agent AOI and the occluder area AOI (the expected ball location) in the final 5 s of TB and FB trials (see Figure 1). *Stimulus order viewed* had no significant impact on mean total looking time (agent area AOI, *F*[1, 33] = 1.75, *p* = .195; occluder area AOI, *F*[1, 33] = 0.024, *p* = .879) or mean total number of fixations (agent area AOI, *F*[1, 33] = 2.21, *p* = .147; occluder area AOI, *F*[1, 33] = 0.70, *p* = .408). Thus, *stimulus order viewed* were collapsed for further analyses.

A second analysis included total looking time and mean number of fixations from the final 5 s of the trial across two levels of the independent variable *Final Outcome* (Ball, No Ball). The analysis revealed no significant effects of *Final Outcome* in AOI in either mean total looking time (agent area AOI, *F*[1, 33] = 3.83, *p* = .541; occluder area AOI, *F*[1, 33] = 0.057, *p* = .813) or mean total number of fixations (agent area AOI, *F*[1, 33] = 1.807, *p* = .189; occluder area AOI, *F*[1, 33] = 0.447, *p* = .509). Hence, *Final Outcome* was collapsed for further analyses.

### 3.2 Main analyses

#### 3.2.1 Preference for looking to false belief over true belief as a proportion of total looking

To examine looking behaviour of infants between age groups, the proportion of the total looking time that occurred during FB trials was calculated. This index was calculated for each participant as follows:

$$\text{proportion}‐\text{to}‐\text{FB index}=\frac{\mathrm{Sum}\;\text{of Looking to}\;\mathrm{FB}\;\text{trials}}{\mathrm{Sum}\;\text{of Looking to}\;\mathrm{FB}\;\text{trials}+\mathrm{Sum}\;\text{of Looking to}\;\mathrm{TB}\;\text{trials}}$$

This index was calculated for (a) total looking time summed across trials by trial type and for (b) total number of fixations summed across trials by trial type with respect to each of the two AOIs: (a) the agent AOI and (b) the occluder area AOI. This looking index allowed us to account for individual variance in baseline looking behaviour between infants (Csibra, Hernik, Mascaro, Tatone, & Lengyel, 2016), and similar indices are used widely across infant literature (Hirshkowitz & Wilcox, 2013; Rutherford, Walsh, & Lee, 2015; Young, Merin, Rogers, & Ozonoff, 2009).

**FIGURE 2:**
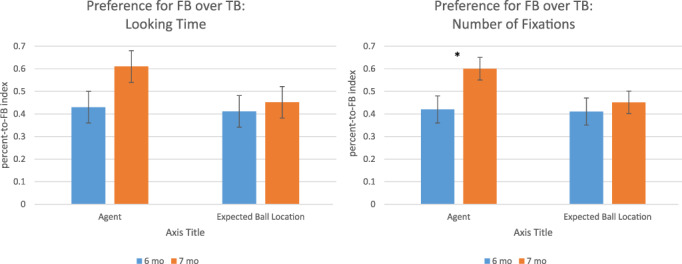
Preference for looking toward false belief trials over true belief trials

An Omnibus repeated‐measures ANOVA was conducted for the proportion‐to‐FB index using total looking time, and another ANOVA was conducted for the proportion‐to‐FB index using number of fixations. First, using proportion‐to‐FB index of number of fixations as a dependent variable, a 2 (AOI: Agent vs. Expected Ball Location) by 2 (Age: 6 vs. 7 months) repeated‐measures ANOVA was conducted. The interaction between the AOI and age was not significant (*F*[1,32] = 1.805, *p* = .19), nor were the main effects of the AOI (*F*[1,32] = 1.516, *p* = .23). The main effect of age was significant for the proportion‐to‐FB index of the number of fixations (*F*[1,32] = 4.087, *p* = .05). Next, using the proportion‐to‐FB index of total looking time as a dependent variable, a 2 (AOI: Agent vs. Expected Ball Location) by 2 (Age: 6 vs. 7 months) repeated‐measures ANOVA was conducted. The interaction between AOI and age was not significant (*F*[1,32] = 1.169, *p* = .29), nor were the main effects of AOI (*F*[1,32] = 1.61, *p* = .21) or age (*F*[1,32] = 2.68, *p* = .11). These relationships are illustrated in Figure 2.

#### 3.2.2 Preference for looking at the agent during false belief trials increased with age

The dependent measures of the proportion‐to‐FB index in summed looking time and, separately, total number of fixations were compared across the between‐subjects factor of *Age* (6 months, 7 months) for each AOI. No significant effects of age were found for the occluder AOI (the expected location of the ball) in total looking time, *F*(1, 33) = 0.508, *p* = .481, and *η*_p_^2^ = 0.016, nor total number of fixations *F*(1, 33) = 0.512, *p* = .479, and *η*_p_^2^ = .016. Significant effects of *Age* were found for the agent AOI in both summed looking time *F*(1, 33) = 4.510, *p* = .042, and *η*_p_^2^ = .124 and total number of fixations *F*(1, 33) = 6.330, *p* = .017, and *η*_p_^2^ = .165, although only the later significance survives comparison to a Bonferroni corrected *α* of .025. The proportion‐to‐FB index to the agent were higher for 7‐month‐olds (*M* = 0.61 for total looking time score and *M* = 0.60 for total number of fixations score) than 6‐month‐olds (*M* = 0.43 for total looking time score and *M* = 0.42 for total number of fixations score).

#### 3.2.3 Do infants show a preference for false or true belief agents?

In addition to testing whether there was development between our 6‐month‐old and 7‐month‐old samples, we could ask whether each group showed a preference for looking to TB or FB displays. We tested whether preference for looking toward FB displays was above chance for each age group. Using the proportion‐to‐FB index for the number of fixations on the agent AOI, 6‐month‐olds were significantly less likely than chance to prefer the FB display (*t*[16] = −1.78. *p* = .047), with 40.5% of their fixations on the FB display. In contrast, 7‐month olds were significantly more likely than chance to prefer the FB display (*t*[16] = 1.94. *p* = .03), with 57.4% of their fixations on the FB display. Using the proportion‐to‐FB index for looking time to the agent AOI, 6‐month‐olds' preference for the TB display was not significant (*t*[16] = −1.40. *p* = .09), with 43% of their fixations on the FB display. Finally, using the proportion‐to‐FB index for looking time, 7‐month‐olds' preference for the FB display was not significant (*t*[16] = 1.39. *p* = .09), with 61% of their fixations on the FB display.

## 4 DISCUSSION

The current study explored infants' looking behaviour across trials that did and did not include an agent with a FB by using an eye‐gaze‐based implicit measure and stimuli adapted from Kovács et al. (2010). Similar to the Kovács et al. study, we found evidence that infants as young as 7 months of age show a different behavioural response to a FB display compared to a TB display, indicating that these infants respond to the agent's true or FBs. While the 7‐month‐olds in the present study looked more to the agent in the final 5 s of FB trials than TB trials, the 6‐month‐olds showed no such preference. Similar results were found using the total looking duration and the total number of fixations. Furthermore, the evidence for elevated interest in FB trials shown in Figure 2 relies on looking toward the agent, regardless of looking toward the more informative location of the ball, suggesting that the infant has linked the FB to the agent who holds the FB. Six‐month‐old participants did not show this tell‐tale pattern of looking times.

Notice that in contrast to other false‐belief tasks, this task does not target the content of the belief, but rather the belief‐holder. We used eye‐tracking technology to measure looking to specific AOSs, the agent or the occluder, rather than looking to the entire display as was measured by Kovács et al. (2010), because we predicted that the agent with a FB would attract the attention of the infant. Seven‐month‐olds fixated the agent AOI in FB trials more than they did in TB trials. This is evidence that 7‐month‐olds will attend to the agent if that agent holds a FB more than if the agent holds a TB. To date, we know of no other evidence that infants bind beliefs or other mental states to the corresponding agent.

Heyes (2014) argued that in Kovács et al.'s original study, results could be explained by the return of the agent interfering with the infant's memory for the movement of the ball to an unseen location. This explanation fails to account for two of the novel findings in the current study. First, it does not explain why looking time specifically *to the agent*, rather than to the display as a whole was greater for FB trials. Second, it does not offer a straight‐forward explanation for why the pattern of looking time across FB and TB trials changed between 6 and 7 months of age.

The current paradigm reveals a measurable response to an agent with a FB as early as 7 months. Prior to Kovács et al. (2010), research examining young infants' understanding of FBs had used experimental tasks embedded with preference information (He, Bolz & Baillargeon, 2012; Luo, 2011). For example, Luo (2011) found that 10‐month‐old infants looked longer when agents grasped a new object over a previously preferred object only when the agent appeared to have knowledge that there were two objects present. In contrast, when the agent knew there was only one object present (TB) or falsely believed there was only one object present (FB), the infants did not expect the agent to have a preference. Similarly, He, Bolz and Baillargeon (2012) found that 10‐11‐month‐old infants looked longer when an agent grasped a short container with a preferred toy collapsed and moved in the agent's absence (FB) than when an agent grasped a tall container without the preferred, long toy. In both cases, the results of these studies suggested infants' sensitivity to FBs involving agent preferences. The stimuli in the current study differ from those used in preference paradigms. In the current study, the agent does not necessarily have a clear goal in mind; rather, he just observes, and one cannot ascribe a clear preference to the agent. The only clues infants might have about the agent's disposition involve his gaze.

In the current study, looking patterns differ between 6‐ and 7‐month‐olds. The 7‐month‐olds looked to the character in the FB trials more than in the TB trials, but the 6‐month‐olds did not. One possible explanation for this difference is that the ability to understand cues to FBs develops between the ages of 6 and 7 months. However, there are other explanations that could account for these findings. Technically, this is not a FB task as the infant is not required to predict the actor's behaviour, so *not* looking at the agent who has a FB does not necessarily mean that the infant does not know that the agent has a FB. It is possible that 6‐month‐olds do not focus on agents preferentially to the rest of the scene despite understanding that the agent has a FB. The gaze pattern of these 6‐month‐olds does not necessarily mean that they have not detected the difference between TB and FB, but could reflect the fact that they do not consider the agent as interesting. Our dependent variable relies on the agent as the region of interest, and a difference in interest in agents generally between 6 and 7 months of age could account for this difference. It is also possible that 6‐month‐olds understood that the character had a FB but did not have a coherent plan for looking for consequences of the FBs. These results are still evidence of socio‐cognitive development, but the current paradigm cannot distinguish between an appreciation of beliefs versus other psychological processes that might be part of such development. Previous research indicates that infants as young as 3–5 months are surprised by objects disappearing (Luo, Kaufman, & Baillargeon, 2009; Wang, Baillargeon, & Paterson, 2005), suggesting the age differences found in this study are not based upon task difficulty related to infants' ability to track the events from their own perspective.

It would be imprudent to conclude that the pattern of results seen in the 6‐month‐old group was clear evidence of immaturity with respect to false‐belief reasoning. Extant research with 6‐month‐olds suggests that they may be sensitive to FBs. Hyde et al. (2018) used near‐infrared spectroscopy (NIRS) to examine infants' functional brain responses in temporal, parietal, and frontal regions, including the temporal–parietal junction (TPJ), an area well known for theory of mind reasoning (Aichhorn et al., 2009; Braukmann et al., 2018; Lloyd‐Fox et al., 2018; Saxe & Kanwisher, 2003; Saxe & Wexler, 2005; Young, Dodell‐Feder, & Saxe, 2010). They found that 6‐month‐olds and 7‐month‐olds show evidence of FB reasoning using a FB task. Researchers found that neural activity in 7‐month‐old infants differentiated only in the TPJ to TB and FB scenarios; the same pattern of results was found in the adult literature. Southgate and Vernetti (2014) used EEG to measure 6‐month‐olds' motor cortex activation by alpha suppression. They found that infants' motor cortex showed activation only when an agent had a FB about a ball being in a box (expecting the agent to reach for the ball), but not when an agent had a FB about the ball not present in the box. This study involves not only tracking belief but predicting behaviour. Neither imaging study reports corroborating eye‐tracking or behavioural data, so it is possible that young infants' processing of FBs is only measurable with neural measurements. Future research could examine both neural and eye‐tracking data together to see whether this is the case.

It is now clear that early estimates of the age of theory of mind development based on the child's explicit report were too conservative. Children do show evidence of theory of mind reasoning much earlier than the often cited 4 years of age, possibly as early as the first year of life. The development of methods that are sensitive enough to measure earlier theory of mind reasoning has some theoretical and potentially some clinical implications. The earliest age of theory of mind reasoning is relevant to any theory that posits such reasoning as a precursor of some other behaviour. For example, Leslie posits that pretend play is evidence of theory of mind development (Leslie, 1992), but this suggestion is troubled by persistent claims that theory of mind abilities develop only at 4 years of age since pretend play is evident as early as 18 months of age. If pretend play depends on theory of mind development and is evident at 18 months of age, then theory of mind cannot develop as late as 4 years of age.

In summary, this study shows evidence of theory of mind reasoning at 7 months of age, and a lack of such evidence at 6 months of age. The fact that 7‐month‐olds looked longer at the observing character who holds a FB when this character had not conveyed any goal or preference suggests that infants has made a link between the FB and the character who holds that FB. Together, these results provide evidence of an appreciation of FB at 7 months and suggest some development between 6 and 7 months.

## CONFLICT OF INTEREST

The authors declare no conflicts of interest.

## ETHICS STATEMENT

Ethics permission for was obtained by the McMaster University Research Ethics Board.

## Data Availability

The data from this study are available from the corresponding author upon reasonable request.

## APPENDIX A.

Means and standard deviations of the proportion‐to‐FB index based on fixation durations (Tables A1 and A2) and number of fixations (Tables A3 and A4) to false belief (FB) and true belief (TB) trials in agent (top) and occluder (bottom) areas of interest (AOIs).

**TABLE A1:** Proportion‐to‐FB index based on fixation durations measured within the agent AOI

Age group	Area of interest							
	Agent: Fixation durations							
	FB 1 *M* (*SD*)	FB 2 *M* (*SD*)	FB 3 *M* (*SD*)	FB 4 *M* (*SD*)	TB1 *M* (*SD*)	TB 2 *M* (*SD*)	TB 3 *M* (*SD*)	TB 4 *M* (*SD*)
6 months	0.51 (0.87)	0.42 (0.50)	0.61 (0.99)	0.36 (0.71)	0.68 (0.58)	0.68 (0.79)	0.42 (0.96)	0.31 (0.54)
7 months	0.70 (0.83)	0.89 (1.07)	0.34 (0.49)	0.71 (0.94)	0.59 (0.77)	0.52 (0.91)	0.34 (0.70)	0.58 (1.26)
Total	0.61 (0.85)	0.65 (0.86)	0.48 (0.79)	0.54 (0.84)	0.64 (0.67)	0.60 (0.84)	0.38 (0.83)	0.45 (0.98)

**TABLE A2:** proportion‐to‐FB index based on fixation durations within the occluder AOI

Age Group	Areas of interest							
	Occluder: Fixation durations							
	FB 1 *M* (*SD*)	FB 2 *M* (*SD*)	FB 3 *M* (*SD*)	FB 4 *M* (*SD*)	TB1 *M* (*SD*)	TB 2 *M* (*SD*)	TB 3 *M* (*SD*)	TB 4 *M* (*SD*)
6 months	0.59 (0.78)	0.43 (0.53)	0.52 (0.90)	0.57 (0.91)	0.82 (1.06)	0.76 (1.00)	0.91 (1.33)	0.43 (0.72)
7 months	1.11 (1.38)	0.64 (0.94)	1.15 (1.36)	0.85 (0.85)	1.19 (1.45)	1.11 (1.87)	0.87 (1.17)	0.82 (1.10)
Total	0.85 (1.13)	0.54 (0.76)	0.83 (1.17)	0.71 (0.88)	1.00 (1.27)	0.94 (1.49)	0.89 (1.24)	0.63 (0.94)

**TABLE A3:** Number of fixations within the agent AOI

Age Group	Area of interest							
	Agent: Number of fixations							
	FB 1 *M* (*SD*)	FB 2 *M* (*SD*)	FB 3 *M* (*SD*)	FB 4 *M* (*SD*)	TB1 *M* (*SD*)	TB 2 *M* (*SD*)	TB 3 *M* (*SD*)	TB 4 *M* (*SD*)
6 months	1.82 (2.13)	2.00 (2.45)	2.00 (2.78)	1.06 (1.48)	2.65 (1.84)	2.29 (1.96)	1.38 (1.96)	0.94 (1.06)
7 months	2.53 (2.65)	3.29 (3.29)	1.44 (1.75)	2.53 (2.55)	2.47 (3.28)	1.53 (2.51)	1.38 (2.70)	1.41 (1.37)
Total	2.18 (2.39)	2.65 (2.93)	1.73 (2.32)	1.79 (2.19)	2.56 (2.62)	1.91 (2.25)	1.38 (2.32)	1.18 (1.24)

**TABLE A4:** Number of fixations within occluder AOI

Age Group	Area of interest							
	Occluder: Number of fixations							
	FB 1 *M* (*SD*)	FB 2 *M* (*SD*)	FB 3 *M* (*SD*)	FB 4 *M* (*SD*)	TB1 *M* (*SD*)	TB 2 *M* (*SD*)	TB1 *M* (*SD*)	TB 2 *M* (*SD*)
6 months	1.94 (2.19)	1.88 (2.37)	1.24 (1.56)	1.47 (1.46)	2.53 (2.40)	2.65 (3.52)	2.56 (3.72)	1.38 (1.89)
7 months	2.06 (2.38)	2.35 (2.89)	2.75 (2.79)	2.12 (1.80)	2.65 (1.93)	1.29 (1.31)	2.06 (2.21)	3.41 (4.78)
Total	2.00 (2.26)	2.12 (2.61)	1.97 (2.34)	1.79 (1.65)	2.59 (2.15)	1.97 (2.70)	2.31 (3.02)	2.42 (3.77)

## References

[icd2263-bib-0001] Aichhorn, M. , Perner, J. , Weiss, B. , Kronbichler, M. , Staffen, W. , & Ladurner, G. (2009). Temporo‐parietal junction activity in theory‐of‐mind tasks: Falseness, beliefs, or attention. Journal of Cognitive Neuroscience, 21(6), 1179–1192. 18702587 10.1162/jocn.2009.21082

[icd2263-bib-0006] Baron‐Cohen, S. , Leslie, A. M. , & Frith, U. (1985). Does the autistic child have a 'theory of mind'?. New York, NY: Routledge/Taylor & Francis Group. 10.1016/0010-0277(85)90022-8 2934210

[icd2263-bib-0007] Bayless, S. J. , Glover, M. , Taylor, M. J. , & Itier, R. J. (2011). Is it in the eyes? Dissociating the role of emotion and perceptual features of emotionally expressive faces in modulating orienting to eye gaze. Visual Cognition, 19(4), 483–510. 24976782 10.1080/13506285.2011.552895 PMC4072640

[icd2263-bib-0008] Braukmann, R. , Lloyd‐Fox, S. , Blasi, A. , Johnson, M. H. , Bekkering, H. , Buitelaar, J. K. , & Hunnius, S. (2018). Diminished socially selective neural processing in 5‐month‐old infants at high familial risk of autism. European Journal of Neuroscience, 47(6), 720–728. 29057566 10.1111/ejn.13751 PMC5943701

[icd2263-bib-0010] Buttelmann, F. , Suhrke, J. , & Buttelmann, D. (2015). What you get is what you believe: Eighteen‐month‐olds demonstrate belief understanding in an unexpected‐identity task. Journal of Experimental Child Psychology, 131, 94–103. 25544393 10.1016/j.jecp.2014.11.009

[icd2263-bib-0013] Csibra, G. , Hernik, M. , Mascaro, O. , Tatone, D. , & Lengyel, M. (2016). Statistical treatment of looking‐time data. Developmental Psychology, 52(4), 521–536. 26845505 10.1037/dev0000083 PMC4817233

[icd2263-bib-0016] He, Z. , Bolz, M. , & Baillargeon, R. (2012). 2.5‐year‐olds succeed at a verbal anticipatory‐looking false‐belief task. British Journal of Developmental Psychology, 30(1), 14–29. 22429030 10.1111/j.2044-835X.2011.02070.x PMC3351383

[icd2263-bib-0049] Heyes, C. (2014). False belief in infancy: A fresh look. Developmental Science, 17(5), 647–659. 24666559 10.1111/desc.12148

[icd2263-bib-0017] Hirshkowitz, A. , & Wilcox, T. (2013). Infants' ability to extract three‐dimensional shape from coherent motion. Infant Behavior & Development, 36(4), 863–872. 24239879 10.1016/j.infbeh.2013.09.003 PMC3882079

[icd2263-bib-0018] Hyde, D. C. , Simon, C. E. , Ting, F. , & Nikolaeva, J. I. (2018). Functional organization of the temporal‐parietal junction for theory of mind in preverbal infants: A near‐infrared spectroscopy study. The Journal of Neuroscience: The Official Journal of the Society for Neuroscience, 38(18), 4264–4274. 29593053 10.1523/JNEUROSCI.0264-17.2018 PMC6596006

[icd2263-bib-0019] Kovács, A. M. , Téglás, E. , & Endress, D. A. (2010). The social sense: Susceptibility to others' beliefs in human infants and adults. Science, 330, 1830–1834. 21205671 10.1126/science.1190792

[icd2263-bib-0020] Leslie, A. (1992). Pretense, autism, and the theory‐of‐mind module. Current Directions in Psychological Science, 1(1), 18–21.

[icd2263-bib-0021] Lloyd‐Fox, S. , Blasi, A. , Pasco, G. , Gliga, T. , Jones, E. H. J. , Murphy, D. G. M. , … Johnson, M. H. (2018). Cortical responses before 6 months of life associate with later autism. European Journal of Neuroscience, 47(6), 736–749. 29057543 10.1111/ejn.13757 PMC5900943

[icd2263-bib-0022] Luo, Y. (2011). Do 10‐month‐old infants understand others' false beliefs? Cognition, 121(3), 289–298. 21861998 10.1016/j.cognition.2011.07.011

[icd2263-bib-0023] Luo, Y. , Kaufman, L. , & Baillargeon, R. (2009). Young infants' reasoning about physical events involving inert and self‐propelled objects. Cognitive Psychology, 58(4), 441–486. 19232579 10.1016/j.cogpsych.2008.11.001 PMC2695492

[icd2263-bib-0024] Mireault, G. C. , Crockenberg, S. C. , Sparrow, J. E. , Pettinato, C. A. , Woodard, K. C. , & Malzac, K. (2014). Social looking, social referencing and humor perception in 6‐and‐12‐month‐old infants. Infant Behavior and Development, 37(4), 536–545. 25061893 10.1016/j.infbeh.2014.06.004 PMC4262602

[icd2263-bib-0025] Neath, K. , Nilsen, E. S. , Gittsovich, K. , & Itier, R. J. (2013). Attention orienting by gaze and facial expressions across development. Emotion, 13(3), 397–408. 23356559 10.1037/a0030463 PMC3925116

[icd2263-bib-0026] Onishi, K. H. , & Baillargeon, R. (2005). Do 15‐month‐old infants understand false beliefs? Science, 8, 255–258. 10.1126/science.1107621 PMC3357322 15821091

[icd2263-bib-0027] Perner, J. , Frith, U. , Leslie, A. M. , & Leekam, S. R. (1989). Exploration of the autistic child's theory of mind: Knowledge, belief, and communication. Child Development, 60(3), 689–700. 2737018

[icd2263-bib-0029] Premack, D. , & Woodruff, G. (1978). Does the chimpanzee have a theory of mind? Behavioral and Brain Sciences, 1(4), 515–526.

[icd2263-bib-0030] Rutherford, M. D. , Walsh, J. A. , & Lee, V. (2015). Brief report: Infants developing with ASD show a unique developmental pattern of facial feature scanning. Journal of Autism and Developmental Disorders, 45(8), 2618–2623. 25703032 10.1007/s10803-015-2396-7

[icd2263-bib-0032] Saxe, R. , & Kanwisher, N. (2003). People thinking about thinking people. the role of the temporo‐parietal junction in "theory of mind". NeuroImage, 19(4), 1835–1842. 12948738 10.1016/s1053-8119(03)00230-1

[icd2263-bib-0033] Saxe, R. , & Wexler, A. (2005). Making sense of another mind: The role of the right temporo‐parietal junction. Neuropsychologia, 43(10), 1391–1399. 15936784 10.1016/j.neuropsychologia.2005.02.013

[icd2263-bib-0034] Southgate, V. , Chevallier, C. , & Csibra, G. (2010). Seventeen‐month‐olds appeal to false beliefs to interpret others referential communication. Developmental Science, 13(6), 907–912. 20977561 10.1111/j.1467-7687.2009.00946.x

[icd2263-bib-0035] Southgate, V. , Senju, A. , & Csibra, G. (2007). Action anticipation through attribution of false belief by 2‐year‐olds. Psychological Science, 18(7), 587–592. 17614866 10.1111/j.1467-9280.2007.01944.x

[icd2263-bib-0036] Southgate, V. , & Vernetti, A. (2014). Belief‐based action prediction in preverbal infants. Cognition, 130(1), 1–10. 24140991 10.1016/j.cognition.2013.08.008 PMC3857687

[icd2263-bib-0037] Surian, L. , Caldi, S. , & Sperber, D. (2007). Attribution of beliefs by 13‐month‐old infants. Psychological Science, 18(7), 580–586. 17614865 10.1111/j.1467-9280.2007.01943.x

[icd2263-bib-0040] Wang, S. H. , Baillargeon, R. , & Paterson, S. (2005). Detecting continuity violations in infancy: A new account and new evidence from covering and tube events. Cognition, 95(2), 129–173. 15694644 10.1016/j.cognition.2002.11.001 PMC3357327

[icd2263-bib-0042] Wellman, H. M. , Cross, D. , & Watson, J. (2001). Meta‐analysis of theory‐of‐mind development: The truth about false belief. Child Development, 72(3), 655–684. 10.1111/1467-8624.00304 11405571

[icd2263-bib-0044] Wimmer, H. , & Perner, J. (1983). Beliefs about beliefs: Representation and constraining function of wrong beliefs in young children's understanding of deception. Cognition, 13(1), 103–128. 6681741 10.1016/0010-0277(83)90004-5

[icd2263-bib-0045] Woodward, A. L. (1998). Infants selectively encode the goal object of an actor's reach. Cognition, 69(1), 1–34. 9871370 10.1016/s0010-0277(98)00058-4

[icd2263-bib-0047] Young, G. S. , Merin, N. , Rogers, S. J. , & Ozonoff, S. (2009). Gaze behavior and affect at 6 months: Predicting clinical outcomes and language development in typically developing infants and infants at risk for autism. Developmental Science, 12(5), 798–814. 19702771 10.1111/j.1467-7687.2009.00833.x PMC2732664

[icd2263-bib-0048] Young, L. , Dodell‐Feder, D. , & Saxe, R. (2010). What gets the attention of the temporo‐parietal junction? an fMRI investigation of attention and theory of mind. Neuropsychologia, 48(9), 2658–2664. 20470808 10.1016/j.neuropsychologia.2010.05.012

